# Consequences of Medium‐Pore Zeolite Constraints for Alkene Cracking—The Case of *n*‐Pentene

**DOI:** 10.1002/anie.4217298

**Published:** 2026-04-30

**Authors:** Ruixue Zhao, Stefan Schallmoser, Gary L. Haller, Maricruz Sanchez‐Sanchez, Johannes A. Lercher

**Affiliations:** ^1^ Department of Chemistry TUM School of Natural Sciences Catalysis Research Center Technical University of Munich Garching Germany; ^2^ Department of Chemical and Environmental Engineering Yale University New Haven Connecticut USA; ^3^ Institute of Chemical Environmental and Bioscience Engineering Vienna University of Technology Vienna Austria

**Keywords:** alkene cracking, confinement, reaction kinetics, transition‐state stabilization, zeolite

## Abstract

The catalytic cracking of alkenes in zeolites is of fundamental and industrial significance, yet the elementary steps of the mechanism are surprisingly less well established than those of alkane cracking. Here, pentenes were employed as model alkenes to investigate cracking kinetics and pathways on H‐ZSM‐5 (**MFI** framework) at 703–843 K. Cracking is initiated from a hydrogen‐bonded alkene, with specific carbenium ions acting as transition states or short‐lived intermediates. Monomolecular cracking, quantified via ethene formation, has an intrinsic activation enthalpy (Δ*H* ^ǂ^°_int._) of 167 kJ·mol^−1^, which is 26 kJ·mol^−1^ lower than for *n*‐pentane, while maintaining comparable activation entropies (−3 vs. 3 J·mol^−1^·K^−1^), resulting in a 28‐fold higher activity at 773 K. Butene formation follows two temperature‐dependent pathways: dimerization cracking via tertiary‐to‐secondary carbenium ions at 703–733 K (Δ*H* ^ǂ^°_int._ = 64 kJ·mol^−1^) and monomolecular cracking involving CH_3_^+^ formation at 813–843 K (Δ*H* ^ǂ^°_int._ = 184 kJ·mol^−1^). Extending the analysis to other medium‐pore zeolite frameworks such as **TON** and **FER** demonstrates that narrower pore systems suppress activity by increasing Δ*H* ^ǂ^°_int._, whereas extra‐framework aluminum oxide promotes reactivity by entropically shifting the transition state to a later stage. Together, these results establish alkene cracking in zeolites as an enthalpy–entropy–controlled process dictated by topology and local chemical environment.

## Graphical abstract

Alkene cracking in medium‐pore zeolites is governed by a balance of enthalpic and entropic effects. Intrinsic barriers are quantified and shown to be lower than for alkanes due to stabilization of carbenium‐ion‐like transition states. Confinement and extra‐framework aluminum modulate reactivity by tuning transition‐state energetics, providing a framework for understanding and controlling cracking reaction.

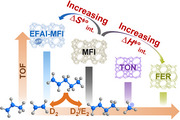

## 1 Introduction

The transformation of alkenes on zeolites, passing through carbenium ion states, tends to involve a multitude of reaction steps, including isomerization, hydride transfer, oligomerization, and cracking [1, 2, 3, 4, 5]. Despite the widespread industrial relevance of these reactions, detailed mechanistic understanding linking the elementary steps to the properties of Brønsted acid sites (BAS) and local reaction environment remains limited [3, 4, 6, 7, 8, 9, 10, 11]. Under cracking conditions, reactions are generally rationalized by transient carbenium‐ion‐like states rather than stable alkoxide intermediates, although direct experimental evidence remains scarce [2, 12].

H‐ZSM‐5 and other medium pore zeolites are industrially established additives to enhance propene formation in fluid catalytic cracking [3, 13, 14, 15, 16, 17, 18]. The three‐dimensional pore structure of H‐ZSM‐5 consists of interconnecting sinusoidal and straight 10‐membered ring channels that host strong Brønsted acid sites (SBAS), which catalyze cracking reactions [2, 4]. Adsorption of short‐chain alkenes on these acid sites is commonly described as the formation of a *π*‐complex followed by proton transfer to the double bond, yet the nature and stability of the resulting surface species under reaction conditions remain debated [19, 20, 21, 22, 23].

Experimental spectroscopic evidence supports the formation of *π*‐complexes upon alkene adsorption, manifested by characteristic shifts of the zeolite bridging hydroxyl vibration [24]. Owing to the high reactivity of alkenes, however, adsorption structures and energetics under cracking conditions have been addressed predominantly by theoretical studies [19, 23, 25, 26, 27, 28, 29]. In a preceding contribution, we demonstrated that linear pentenes adsorb on H‐FER and H‐ZSM‐5 primarily as *π*‐complex, whereas alkoxide species form only via bimolecular routes [24]. These findings underscore the importance of adsorption thermodynamics in governing alkene reactivity.

Cracking of C_4_ to C_6_ alkenes proceeds via both monomolecular and bimolecular (dimerization‐cracking) pathways [7, 8, 9, 11, 30, 31, 32, 33, 34, 35, 36, 37, 38, 39, 40, 41, 42, 43]. While previous studies have provided valuable insight into reaction routes and product selectivities, quantitative experimental information on intrinsic activation barriers for individual alkene cracking steps remains scarce, with only a few studies directly addressing *β*‐scission energetics under catalytic conditions [4, 32, 44, 45, 46, 47]. Moreover, the origin of the substantially higher intrinsic activity of alkene cracking compared to alkane cracking has not been unequivocally resolved.

In addition to the specific reaction pathways, the local zeolite environment strongly influences cracking reactivity. For alkane cracking, both pore confinement and extra‐framework species located near BAS have been shown to modulate rates by altering enthalpic and entropic contributions to the transition state [48, 49, 50, 51, 52, 53, 54]. Whether similar effects govern alkene cracking, and how confinement and active‐site modification jointly shape intrinsic barriers, remain open questions [55, 56, 57].

Here, we investigate the cracking of linear pentenes as a model system to elucidate the elementary steps, intrinsic activation barriers, and environmental effects governing alkene cracking in medium‐pore zeolites. By combining kinetic analysis with adsorption thermodynamics, and by comparing **MFI**, **TON**, and **FER** frameworks, as well as extra‐framework aluminum modified catalysts, we provide a quantitative framework linking alkene cracking reactivity to confinement and active‐site environments.

## 2 Results and Discussion

### 2.1 Reaction Network of Pentene Cracking

**SCHEME 1:**
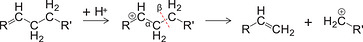
Illustration of alkene cracking via carbenium ions following the empirical *β*‐scission rule.

Alkene cracking on acidic zeolites is rationalized within a carbenium‐ion‐mediated *β*‐scission framework [3, 4, 6, 7, 8, 9, 10, 11, 32, 44, 45, 46, 47]. In this representation cleavage occurs at the carbon–carbon bond in the *β*‐position relative to the positive charge, generating a free alkene and a transient carbenium‐ion configuration (Scheme 1). The latter may subsequently desorb to form a second alkene, undergo further *β*‐scission, or participate in alkylation with another alkene.

In Scheme 1, the carbenium ion should be understood not as a stable surface intermediate, but as a higher lying transition‐state configuration in which the C─C bond in the *β*‐position is weakened. This implies that specific primary, secondary, and tertiary carbenium ions must exist in the reaction path. This hypothesis will be revisited in connection with Scheme 3 in the final section.

**FIGURE 1:**
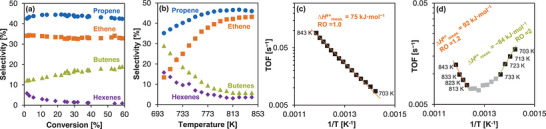
Distribution of the main products over MFI‐90 as a function of (a) pentene conversion at 763 K and (b) reaction temperature at low conversions of 2%–5%. (● = propene, ■ = ethene, ▲ = butenes, ◆ = hexenes; conditions: *W*_cat._/*F*_pentene_ = 0.2–7.0 g·s·cm^−3^.) Temperature dependence of (c) ethene and (d) butene formation rates during pentene cracking over MFI‐90 (pentene conversion ≤ 5%). Turnover frequencies (TOFs) are plotted on a logarithmic scale as Arrhenius‐type plots. TOFs were determined under differential conditions at 703–843 K by normalizing the reaction rates to the number of SBAS. Activation energies were extracted using the Eyring equation, as described in Supporting Information, Section S3.5. Additional details on the definition and calculation of RO are provided in Figure S8.

Cracking of pentenes was studied in the temperature range of 703–843 K on MFI‐90 (physicochemical properties summarized in Table S1). Under these reaction conditions, rapid alkene isomerization leads to a quasi‐equilibrated distribution of pentene isomers, allowing them to be treated collectively in the kinetic analysis (Supporting Information Section S3.2). Ethene, propene, butenes, and hexenes comprised about 95% of all products (Figure 1), with cyclopentane formed in smaller amounts as a further significant product (approximately 3%). Trace quantities of pentanes, methane, higher alkenes, aromatics, and H_2_ were also detected. For mechanistic analysis, only the major products are considered.

Figure 1a depicts the dependence of product selectivities on pentene conversion. The selectivity to butenes increased from initially 12% to almost 20% at 60% conversion, accompanied by a decrease in hexene selectivity from 7% to nearly zero. In contrast, ethene selectivity decreased only slightly from 34.5% to 32.5%, while propene selectivity passed through a broad maximum of 44.5% at approximately 15% conversion.

The shift in product distribution at higher conversions, especially the increase of butene selectivity, is attributed to secondary reactions involving addition of pentene isomers to ethene and propene formed as primary products (Scheme S1). The formed higher alkene intermediates (C_7_^=^ to C_10_^=^) subsequently crack to butenes, while ethene and propene species persist at steady‐state concentrations. Alkenes containing more than six carbon atoms were not observed in significant amounts, consistent with their rapid cracking at these temperatures, which prevents accumulation to detectable levels.

Temperature‐dependent experiments conducted at low initial conversions (2%–5%, Figure 1b) reveal a preferential formation of ethene and propene above 723 K. At temperatures exceeding 773 K, these products account for more than 80% of the total product slate. Selectivities associated with dimerization cracking pathway dropped drastically with increasing temperature, that is, butene (hexene) selectivity drops from 29% (16%) at 703 K to 6% (4%) at 843 K, reflecting the thermodynamic disfavoring of dimerization at higher temperatures. Product selectivities approach nearly constant values above 798 K, with propene exhibiting a broad maximum near 813 K.

Considering R1 and R2 as the primary pathways to the major products, selectivities toward butenes and hexenes, as well as to ethene and propene, would be expected to vary in parallel. However, the product distributions (Figure 1) deviate from this stoichiometric expectation over a wide range of temperatures and conversions. For instance, at 763 K and low conversions, the selectivity to propene (*S*_propene_) is 8.5% higher than to ethene (*S*_ethene_), and butene (*S*_butene_) is 5% higher than that of hexene (*S*_hexene_), yielding a ratio close to 2:1. At low conversions, these deviations are satisfactorily explained by secondary cracking of hexene to propene (R4), which consumes hexene (but not butene) and generates additional propene without affecting ethene formation.

(R1) $$C_{5}H_{10}\to C_{2}H_{4}+C_{3}H_{6}\to \left(\mathrm{Reaction}\;1,D_{2}\right)$$

(R2) $$2\;C_{5}H_{10}\to C_{10}H_{20}\to C_{4}H_{8}+C_{6}H_{12}\;\;\left(\mathrm{Reaction}\;2,B_{2}\right)$$

(R3) $$C_{5}{H_{11}}^{+}\to C_{4}H_{8}+H_{3}C^{+}\to \left(\mathrm{Reaction}\;3,D_{2}^{\prime }/E_{2}\right)$$

(R4) $$C_{6}H_{12}\to 2\;C_{3}H_{6}\to \left(\mathrm{Reaction}\;4\right)$$

Accordingly, the rate of ethene formation is used to evaluate the rate of monomolecular cracking, while the rate of butene formation serves as a probe for bimolecular (dimerization) cracking. Contributions from alternative monomolecular routes to butene formation (R3), especially at high temperatures, are discussed in the following section.

### 2.2 Intrinsic Barriers of Alkene Cracking

In the temperature range 703–843 K, ethene formation exhibits a reaction order close to one, consistent with its assignment to monomolecular pentene cracking (type D_2_, reaction orders were determined from the dependence of the reaction rate on pentene partial pressure under differential conditions, details of the fitting are provided in Supporting Information Section S3.2 and Figure S8). The apparent activation enthalpy determined from Eyring equation is Δ*H* ^ǂ^°_meas._ = 75 kJ·mol^−1^ (Figure 1c, Table 1). For comparison, the kinetics of protolytic cracking of *n*‐pentane were measured on MFI‐90 under analogous conditions, and the results are summarized in Table 1.

**TABLE 1:** Overview of apparent and intrinsic activation energies (calculated with Eyring equation), reaction order (RO) and rate constants determined for the different pathways in cracking of pentenes (upper section) and protolytic *n*‐pentane cracking (lower section) measured on catalyst MFI‐90.

Pathway	*T* (K)	Δ*H* ^ǂ^°_meas._ (kJ·mol^−1^)	Δ*S* ^ǂ^°_meas._ (J·mol^−1^·K^−1^)	RO [‐]	*k*_meas._(mol·mol_SBAS_^−1^·s^−1^·bar^−1^)
Monomolecular cracking (type D_2_)	703–843	75 ± 4	−155 ± 6	1	0.54^a^ 1.1^b^ 2.1^c^
Dimerization (type B_2_)	703–733	−84 ± 4	−345 ± 12	2	14.5^a^ ^,*^
Monomolecular cracking type D_2_’	813–843	92 ± 4	−151 ± 8	1.2	0.29^c^
*n*‐Pentane cracking	753–793	128 ± 4	−114 ± 6	1	0.04^b^, ^d^

^a^ At 733 K.

^b^ At 773 K.

^c^ At 813 K.

^d^ Sum of all cracking pathways as determined for *n*‐pentane cracking.

*unit in [mol·mol_SBAS_^−1^·s^−1^·bar^−2^]; ROs were determined from the dependence of the reaction rate on pentene partial pressure (10–60 mbar) under differential conditions, details of the fitting are provided in Figure S8.

In contrast, butene formation originates from two distinct pathways whose contributions vary with temperature. At 703–733 K, butenes are formed predominantly via dimerization cracking (type B_2_), characterized by a reaction order of approximately two. At higher temperatures (813–843 K), monomolecular cracking (type D_2_’/E_2_) contributes to butene formation, resulting in a reaction order approaching one (Figure 1d, Table 1). Accordingly, the Arrhenius‐type plot for butene formation (Figure 1d) reflects a clear transition between these pathways. Dimerization cracking (B_2_, 703–733 K) exhibits a negative apparent activation energy (Δ*H* ^ǂ^°_meas._ = −84 kJ·mol^−1^), whereas monomolecular cracking (D_2_’/E_2_, 813–843 K) shows a positive apparent activation energy of 92 kJ·mol^−1^.

The ground state for monomolecular pentene cracking may, in principle, correspond to either a chemisorbed alkoxide or a *π*‐complex. While earlier mechanistic models commonly assumed alkoxide intermediates at cracking temperatures [44, 47, 58, 59, 60], recent computational studies combining static DFT and molecular dynamics simulations have demonstrated that alkoxides are thermodynamically disfavored under reaction conditions, with linear alkenes (C_5_–C_8_) preferentially existing as *π*‐complexes or weakly physisorbed species [28, 45, 61]. Advanced AIMD simulations by Ren et al. further identified *π*‐complex as kinetically relevant intermediate for alkene cracking [29]. High‐level quantum chemical calculations showed that although C_5_ alkoxides are most stable at 0 K, entropic contributions at elevated temperatures (e.g., 623 K) reverse the stability order, rendering the trans‐2‐pentene *π*‐complex thermodynamically dominant under cracking‐relevant conditions [12]. These findings are consistent with the present experimental observation on MFI.

**SCHEME 2:**
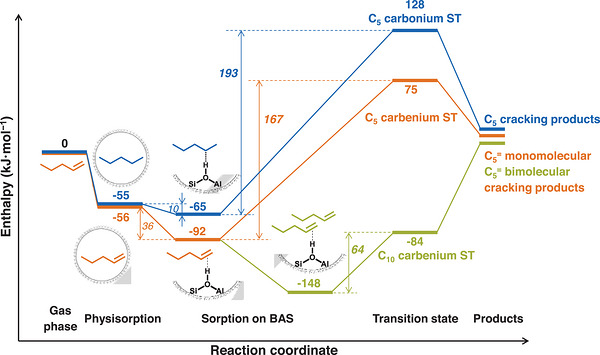
The reaction coordinate diagram with enthalpy changes for pentene and pentane cracking.

On this basis, the π‐complex is taken as the ground state for monomolecular pentene cracking on MFI zeolite. Using previously determined adsorption enthalpies for pentene on H‐ZSM‐5 (physisorption Δ*H*°_phys._ = −56 kJ·mol^−1^, chemisorption (alkoxide) Δ*H*°_chem._ = −133 kJ·mol^−1^, π‐complex Δ*H*°_π_ = −92 kJ·mol^−1^) [24], the intrinsic activation enthalpy can be obtained by correcting the measured apparent enthalpy with Δ*H*°_π_. This yields an intrinsic enthalpy barrier of Δ*H* ^ǂ^°_int._ = 167 kJ·mol^−1^ (Scheme 2, Δ*H* ^ǂ^°_int._ = Δ*H* ^ǂ^°_meas._ − Δ*H*°_π_ = 75 − (−92) = 167 kJ·mol^−1^). This value is substantially lower than the intrinsic barrier for *n*‐pentane cracking (≈ 200 kJ·mol^−1^), determined experimentally and theoretically [44, 46, 47], and also lower by about 40 kJ·mol^−1^ than a hypothetical alkoxide‐mediated pathway for pentene cracking [24]. Such a high barrier would be incompatible with the experimentally observed higher reactivity of pentene relative to pentane, corroborating the hypothesis that the initial state is a π‐complex.

For bimolecular cracking, the most probable initial state consists of a *π*‐complex interacting with a physisorbed pentene molecule, corresponding to a combined adsorption enthalpy of Δ*H*°_dimer._ = −148 kJ·mol^−1^. Correcting the measured barrier accordingly yields an intrinsic activation enthalpy of 64 kJ·mol^−1^ for the bimolecular cracking pathway (Scheme 2, Δ*H* ^ǂ^°_int._ = Δ*H* ^ǂ^°_meas._ − Δ*H*°_dimer._ = −84 − (−148) = 64 kJ·mol^−1^).

At high temperatures (813–843 K), ethene and propene together account for 81%–90% of the product distribution, indicating that bimolecular cracking becomes strongly disfavored. This behavior is attributed to the large entropy penalty associated with dimerization, which dominates the free energy barrier at elevated temperatures. Under these conditions, butene formation increasingly proceeds via a monomolecular pathway involving cleavage of a C_5_ carbenium ion into butene and a CH_3_^+^ (R3). The apparent activation enthalpy for this pathway is 92 kJ·mol^−1^, corresponding to an intrinsic enthalpic barrier of 184 kJ·mol^−1^ after correction with *π*‐complex ground state. This value exceeds that of R1 by 17 kJ·mol^−1^ (Table 1), consistent with the formation of the energetically disfavored CH_3_^+^, which subsequently reacts with excess pentene to form hexene. The dominant monomolecular cracking pathway is type D_2_, (secondary‐to‐primary carbenium ion), while dimerization cracking proceeds primarily via a B_2_‐type *β*‐scission (tertiary‐to‐secondary carbenium ions). Although B‐type cracking pathways are generally expected to be faster due to their lower intrinsic barriers [32, 44, 47], the experimentally observed rate ratio (*r*_dimerization‐cracking_ / *r*_monomolecular‐cracking_ ≈ 27 at 733 K) is significantly lower than predicted by previous models [4]. This discrepancy is attributed to confinement effects in ZSM‐5, which restrict the number of accessible cracking modes for bulky C_10_ carbenium‐ion transition state and increase their effective activation barrier, consistent with theoretical predictions on the influence of substitution and steric constraints on cracking energetics [47].

### 2.3 Comparison of Kinetic Parameters for Alkane and Alkene Cracking

Due to the complexity of the butene formation pathways, the comparison between alkane and alkene cracking is based on ethene formation via the monomolecular D_2_ pathway. As shown in Table 1, the apparent rate constant for monomolecular pentene cracking (*k*C_5_^═^ crack) on MFI‐90 at 773 K is approximately 28 times higher than that for *n*pentane cracking ($k_{C_{5}\mathrm{crack}}$, summing all cracking pathways). This ratio is in good agreement with previously reported values [4]. Since apparent rate constants reflect both ground‐state and transition‐state contributions through the Gibbs free energy of activation, identification of the relevant ground‐state energy is required to assess intrinsic activity differences.

Adsorption thermodynamics of pentene were previously determined by calorimetry on H‐FER [24], yielding $\Delta H_{\mathrm{ads}.C_{5}^{=}}^{\circ }$ = −92 kJ·mol^−1^ and $\Delta S_{\mathrm{ads}.C_{5}^{=}}^{\circ }$ = −182 J·mol^−1^·K^−1^, respectively (Table 2) [24]. Direct measurement of these quantities on H‐MFI is not feasible due to rapid pentene dimerization even at ambient temperature. For *n*‐pentane, however, adsorption enthalpies on H‐FER, H‐TON, and H‐MFI are nearly identical (Δ*H*°_ads._ = −68 ± 4 kJ·mol^−1^), as established in our previous studies (Table 2) [50, 54, 62, 63, 64]. In contrast, adsorption entropies become progressively less negative with increasing pore size, reflecting the reduced confinement.

**TABLE 2:** Adsorption properties of *n*‐pentane and *n*‐pentene in H‐FER, H‐TON, and H‐MFI at 323 K.

Adsorbate	Adsorbent	Δ*H*°_ads._ (kJ·mol^−1^)	Δ*S*°_ads._ (J·mol^−1^·K^−1^)	Δ*G*°_ads._ ^a^ (kJ·mol^−1^)
Pentane (C_5_)	H‐FER	−69^b^	−147^c^	−20
	H‐TON	−71^b^	−138^c^	−25
	H‐MFI	−65^d^	−117^d^	−27
Pentene (C_5_^═^)	H‐FER	−92^e^	−182^e^	−33
	H‐TON	−92^f^	−173^g^	−36
	H‐MFI	−92^e^	−152^g^	−43

^a^ Calculated from Δ*G*°_ads._ = Δ*H*°_ads._ − *T*Δ*S*°_ads. _.

^b^ Adapted from Ref. [65].

^c^ Calculated from the Henry's constant in Ref. [65].

^d^ Average number from Refs. [50, 54, 62].

^e^ Adapted from Ref. [24].

^f^ Assuming the adsorption enthalpy are the same between the different frameworks according to the constant adsorption enthalpy for pentane in FER, TON, and MFI.

^g^ Calculated by $\Delta S_{\mathrm{ads}.C_{5}^{=}}^{\circ }$ (H‐FER) − $\Delta S_{\mathrm{ads}.C_{5}}^{\circ }$ (H‐FER) = $\Delta S_{\mathrm{ads}.C_{5}^{=}}^{\circ }$ (H‐TON) − $\Delta S_{\mathrm{ads}.C_{5}}^{\circ }$ (H‐TON) =$\Delta S_{\mathrm{ads}.C_{5}^{=}}^{\circ }$ (H‐MFI) − $\Delta S_{\mathrm{ads}.C_{5}}^{\circ }$ (H‐MFI).

The invariance of adsorption enthalpy across the three frameworks indicates that interaction strengths between *n*‐pentane and SBAS are primarily governed by intrinsic molecular properties. In other words, for a given adsorbate, the interaction strength changes very little across these three frameworks. By analogy, pentene adsorption is expected to exhibit a similar framework‐independent interaction strength, consistent with the constant adsorption enthalpy of approximately −92 kJ·mol^−1^ [50]. The more exothermic adsorption of pentene relative to pentane arises from additional π‐bonding rather than only dispersion interactions and is accompanied by an additional entropy loss of −35 J·mol^−1^·K^−1^ (−147 vs. −182 J·mol^−1^·K^−1^), reflecting reduced molecular mobility upon π‐complex formation. Assuming this entropy offset to be framework‐independent allows estimation of pentene adsorption entropies in H‐TON and H‐MFI from the corresponding pentane (Table 2).

Using the apparent activation parameters in Table 1 and the adsorption thermodynamics in Table 2, intrinsic activation barriers for pentane and pentene cracking on H‐MFI can be determined. As shown in Table 3, the intrinsic standard Gibbs free energy of activation (Δ*G* ^ǂ^°_int._) for pentene cracking at 773 K is 22 kJ·mol^−1^ lower than that for pentane cracking. Since the difference in adsorption Gibbs free energy at reaction temperature is negligible (approximately 1 kJ·mol^−1^ at 773 K), the lower Δ*G* ^ǂ^°_int._ for pentene cracking is attributed to the greater stabilization of the carbenium‐ion‐like transition state compared to the carbonium‐ion‐like transition state involved in pentane cracking.

**TABLE 3:** Overview of intrinsic energies of pentene and *n*‐pentane cracking on catalyst MFI‐90 at 773 K.

Reaction	Δ*H* ^ǂ^°_int._ (kJ·mol^−1^)	Δ*S* ^ǂ^°_int._ (J·mol^−1^·K^−1^)	Δ*G* ^ǂ^°_int._ (kJ·mol^−1^)
Pentene cracking (mono‐cracking, type D_2_)	167 ± 4	−3 ± 8	169 ± 7
Pentane cracking	193 ± 4	3 ± 6	191 ± 6

The intrinsic activation entropies for pentene and pentane cracking are similar (−3 vs. 3 J·mol^−1^·K^−1^, Table 3), indicating that the enhanced reactivity of pentene cracking is governed primarily by enthalpic effects. Specifically, the intrinsic activation enthalpy for pentene cracking is 26 kJ·mol^−1^ lower than that for *n*‐pentane cracking (Scheme 2, Table 3), corresponding to a 22 kJ·mol^−1^ reduction in the intrinsic standard Gibbs free energy barrier. This difference predicts an intrinsic rate enhancement of approximately 30‐fold at 773 K, in excellent agreement with the experimentally observed rate increase of about 28‐fold.

### 2.4 Impact of Constraints due to Different Zeolite Frameworks

Having identified the intrinsic origin of the higher reactivity of pentene relative to pentane on H‐MFI, we now extend the analysis to assess how pore topology and confinement modulate adsorption thermodynamics and cracking kinetics across different zeolite frameworks. A quantitative understanding of *n*‐pentane adsorption thermodynamics provides the foundation for analyzing pentene adsorption in H‐MFI, H‐TON, and H‐FER zeolites, as both adsorbates experience similar confinement effects within the zeolite pore systems. As summarized in Table 2, adsorption enthalpies of *n*‐pentane are nearly identical across all three frameworks (Δ*H*°_ads._ = −65 to −71 kJ·mol^−1^), indicating comparable van der Waals interactions with the pore walls. In contrast, adsorption entropies (Δ*S*°_ads._) show a systematic dependence on pore topology. The three‐dimensional channel system of H‐MFI permits greater molecular freedom, resulting in the least negative Δ*S*°_ads._ (−117 J·mol^−1^·K^−1^), whereas the one‐dimensional channels of H‐TON and more confined two‐dimensional channels of H‐FER increasingly restrict molecular motion, resulting in Δ*S*°_ads._ values of −138 and −147 J·mol^−1^·K^−1^, respectively. Crucially, the Gibbs free energy of adsorption (Δ*G*°_ads._) becomes progressively less favorable with increasing confinement (Δ*G*°_ads._: H‐MFI < H‐TON < H‐FER).

Building upon this framework, pentene exhibits analogous but more pronounced adsorption behavior. The adsorption enthalpy of pentene is essentially invariant across H‐MFI, H‐TON, and H‐FER (Table 2, Δ*H*°_ads_ = −92 kJ·mol^−1^), reflecting similar interaction strengths with the zeolites. However, the entropic penalty associated with adsorption increases systematically with confinement trend observed for *n*‐pentane, with strongest stabilization in H‐MFI and weakest in H‐FER. The more favorable adsorption of pentene relative to pentane arises from additional *π*‐interactions with SBAS, while preserving the fundamental enthalpy‐entropy balance imposed by pore topology.

**FIGURE 2:**
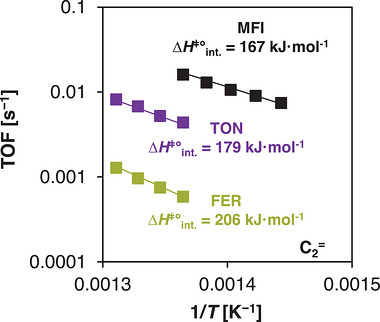
Temperature dependence of ethene formation rate in MFI‐90, H‐TON, and H‐FER. TOFs are plotted on a logarithmic scale as Arrhenius‐type plots.

The rate of pentene cracking, quantified via ethene formation via D_2_ pathway, decreases in the order H‐MFI > H‐TON > H‐FER (Figure 2). From temperature‐dependent rate measurements, apparent activation barriers were derived using the Eyring equation (Table 4). Combining these values with the adsorption thermodynamics in Table 2 allows determination of intrinsic activation barriers. Notably, the intrinsic standard Gibbs free energy of activation is nearly identical for all three frameworks (Δ*G* ^ǂ^°_int._ ≈ 165 ± 4 kJ·mol^−1^), indicating strong enthalpy‐entropy compensation across the different pore topologies.

**TABLE 4:** Overview of energy barriers of pentene cracking on MFI, TON, and FER catalysts at 733 K.

Catalysts	Δ*H* ^ǂ^°_meas._ (kJ·mol^−1^)	Δ*S* ^ǂ^°_meas._ (J·mol^−1^·K^−1^)	Δ*G* ^ǂ^°_meas._(kJ·mol^−1^)	Δ*H* ^ǂ^°_int._(kJ·mol^−1^)	Δ*S* ^ǂ^°_int._ (J·mol^−1^·K^−1^)	Δ*G* ^ǂ^°_int._ (kJ·mol^−1^)
H‐FER	114 ± 5	−128 ± 6	208 ± 7	206 ± 5	54 ± 6	167 ± 7
H‐TON	87 ± 5	−149 ± 8	196 ± 8	179 ± 5	24 ± 8	161 ± 8
H‐MFI	75 ± 4	−155 ± 9	189 ± 8	167 ± 4	−3 ± 9	169 ± 8

This compensation implies that zeolite confinement shifts the standard free energies of both the adsorbed ground state and the carbenium‐ion‐like transition states in a correlated manner. Increasing confinement imposes a stronger entropic penalty on adsorption, which leads to higher apparent activation barriers in narrower pore systems. Thus, after correcting for adsorption thermodynamics, however, the intrinsic standard free‐energy barrier for C–C bond scission varies only weakly across **MFI**, **TON**, and **FER**, demonstrating that the elementary cracking step itself is largely framework‐independent.

### 2.5 Impact of Constraints due to the Presence of Extra‐Framework Aluminum (EFAl) on Cracking of Pentenes

While framework topology primarily modulates pentene cracking through enthalpy‐entropy compensation associated with confinement, additional constraints arise when extra‐framework aluminum (EFAl) species are present in proximity to SBAS. In our previous investigations on pentane cracking, EFAl was shown to induce a pronounced entropic shift toward a later transition state, leading to substantial rate enhancement [50, 53, 54]. Whether this effect is specific to alkane cracking or represents a more general phenomenon remains unclear. Here, we extend this analysis to pentene cracking

**FIGURE 3:**
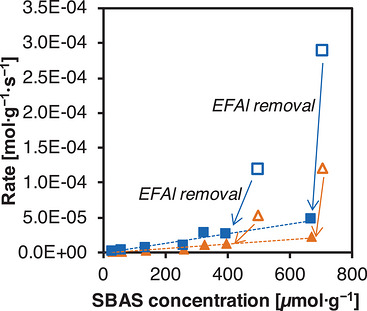
Formation rates of ethene (blue squares) and butene (orange triangles) as a function of SBAS concentration at 763 K for a series of MFI samples (properties listed in Table S1). Solid symbols represent samples exhibiting linearity, that is, constant cracking rate per SBAS. Open symbols correspond to high‐Al‐content samples prior to AHFS modification showing elevated rates. Arrows indicate the effect of the AHFS treatment.

Figure 3 shows that, for MFI samples with low SBAS concentrations (< 400 µmol·g^−1^), the pentene cracking rate increases linearly with SBAS concentration, consistent with all SBAS being equally active. This behavior mirrors that observed for *n*‐pentane cracking and confirms that isolated SBAS govern the intrinsic activity of alkene cracking in the absence of EFAl [50]. At higher Al contents, however, clear deviations from linearity are observed. These samples (MFI‐15 and MFI‐25) contain significant fractions of EFAl species located in proximity to SBAS (EFAl‐SBAS), as established previously by infrared spectroscopy of adsorbed base molecules (Supporting Information, Section S2.3) [50]. The elevated cracking rates observed for these materials indicate that EFAl modifies the local reaction environment of adjacent SBAS.

Selective removal of EFAl species by ammonium hexafluorosilicate (AHFS) treatment restores a constant cracking rate per SBAS, identical to that of low‐Al MFI samples (Figure 3). This behavior is observed for both monomolecular and biomolecular pentene cracking pathways and closely parallels earlier findings for pentane cracking [50]. These results demonstrate that the enhanced activity originates from EFAl‐SBAS ensembles rather than from changes in the intrinsic reactivity of isolated SBAS.

**FIGURE 4:**
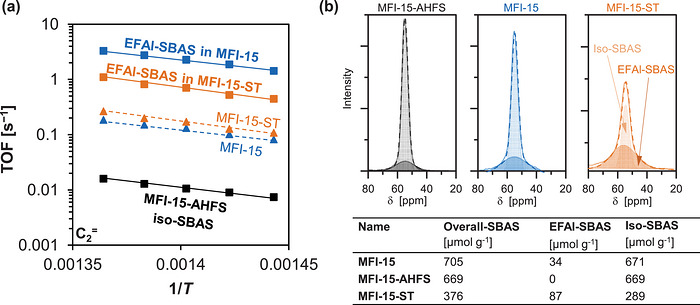
(a) Temperature dependence of ethene formation rates over MFI‐15‐AHFS (black square, also representing iso‐SBAS), MFI‐15, (blue triangle), and MFI‐15‐ST (orange triangle), together with the deconvoluted rates associated with EFAl‐SBAS in MFI‐15 (blue square) and MFI‐15‐ST (orange square). (b) 1D ^27^Al MAS NMR spectra and the concentrations of overall SBAS, iso‐SBAS, and EFAl‐SBAS in the MFI‐15 series adapted from ref. [50]. TOFs are plotted on a logarithmic scale as Arrhenius‐type plots. TOFs for MFI‐15‐AHFS, MFI‐15, and MFI‐15‐ST were normalized to the concentration of overall SBAS, while TOFs for EFAl‐SBAS were calculated according to Equation 1. Reaction condition: *m*_cat._ = 1–5 mg (250–315 µm), charged with SiC dilution (250–315 µm, 299–295 mg); *p*_pentene_ = 26.42 mbar; N_2_ carrier, total flow = 400 mL·min^−1^; pentene conversion ≤ 5%. Figure (b) is adapted with permission from Elsevier, 2014 Elsevier Inc.

To quantify the effect of EFAl, the MFI‐15 sample was systematically modified to vary the fraction of EFAl‐SBAS: AHFS treatment removed EFAl species (MFI‐15‐AHFS), while steaming increased their abundance (MFI‐15‐ST). Pentene cracking rates on MFI‐15 and MFI‐15‐ST exceed those on MFI‐15‐AHFS by more than an order of magnitude (Figure 4). Apparent activation parameters for monomolecular pentene cracking (D_2_) are summarized in Table 5. The AHFS‐treated sample exhibits an apparent activation enthalpy identical to that of low‐Al MFI (Table 1, MFI‐90, 75 kJ·mol^−1^), whereas EFAl‐containing samples display slightly high activation enthalpies, consistent with earlier observations for alkane cracking [50, 53]. Importantly, this increase is more than compensated by a substantial rise in activation entropy, resulting in markedly lower apparent free‐energy barriers.

**TABLE 5:** Overview of energy barriers of pentene cracking on MFI‐15 samples, iso‐SBAS, and EFAl‐SBAS determined from different samples at 733 K.

	Δ*H* ^ǂ^°_meas._ (kJ·mol^−1^)	Δ*S* ^ǂ^°_meas._ (J·mol^−1^·K^−1^)	Δ*G* ^ǂ^°_meas._ (kJ·mol^−1^)
MFI‐15‐AHFS (Iso‐SBAS)	75 ± 4	−155 ± 9	189 ± 8
MFI‐15	80 ± 4	−127 ± 8	173 ± 7
MFI‐15‐ST	88 ± 4	−113 ± 8	171 ± 7
EFAl‐SBAS (MFI‐15)	81 ± 5	−102 ± 8	156 ± 8
EFAl‐SBAS (MFI‐15‐ST)	90 ± 4	−100 ± 8	163 ± 7

To disentangle site‐specific effects, turnover frequencies (TOFs) were therefore deconvoluted into contributions from isolated SBAS (iso‐SBAS) and EFAl‐enhanced sites (EFAl‐SBAS) using the independently determined concentrations of each site (Equation 1, Supporting Information, Section S2.3) [50]. As shown in Figure 4, EFAl‐SBAS in MFI‐15 exhibits a TOF approximately 200 times higher than that of iso‐SBAS at 733 K (3.30 vs. 0.016 s^−1^). this enhancement arises almost exclusively from a less negative activation entropy (−102 vs. −155 J·K^−1^·mol^−1^), while the apparent activation enthalpy remains comparable (Table 5). These results demonstrate that EFAl accelerates pentene cracking by increasing the transition entropy, which is attributed to a shift of the transition state toward a later configuration, in which the carbon atoms of the breaking bond are further apart.

(1) $$\begin{array}{ccc} & & TOF_{\mathrm{overall}-\mathrm{SBAS}}\times \;c_{\mathrm{overall}-\mathrm{SBAS}}=TOF_{\mathrm{iso}-\mathrm{SBAS}}\;\times c_{\mathrm{iso}-\mathrm{SBAS}}\\ & & +\;TOF_{\mathrm{EFAl}-\mathrm{SBAS}}\times c_{\mathrm{EFAl}-\mathrm{SBAS}} \end{array}$$

where *c*_i_ is the concentration of SBAS of each type in mol·g^−1^.

**FIGURE 5:**
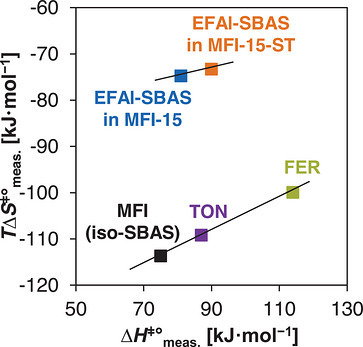
Correlation of measured apparent activation entropy and enthalpy for pentene cracking (ethene formation) on non‐EFAl containing **MFI** (iso‐SBAS), **TON**, **FER,** and EFSi‐SBAS in MFI‐15 and MFI‐15‐ST. (T = 733 K).

The magnitude of EFAl effect depends on the local environment. In MFI‐15‐ST, EFAl‐SBAS shows a smaller rate enhancement (60–70‐fold relative to iso‐SBAS), accompanied by a higher activation enthalpy but a similar activation entropy compared to MFI‐15. This behavior is attributed to extensive dealumination by steaming, which generates EFAl species located within the pores rather than adjacent to SBAS. These species effectively narrow the pores, imposing additional steric constraints analogous to those observed in **TON** and **FER**. As a result, the enthalpic penalty associated with cracking increases, offsetting part of the entropic benefit provided by EFAl, as illustrated by the enthalpy‐entropy correlation in Figure 5.

**SCHEME 3:**
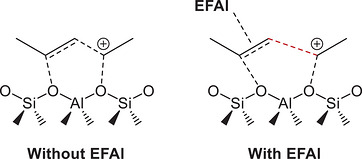
The presumable transition state for cracking of a linear pentene molecule catalyzed by a zeolitic BAS.

Scheme 3 depicts the proposed transition state for monomolecular alkene cracking. Theoretical calculations point toward formation of a six‐ring structure, where two oxygen atoms next to Al are involved [50, 66]. Charge analysis revealed that there is a positive charge located at the hydrocarbon fragment [66]. The transition state is described as a highly polarized entity consisting of a strongly surface‐bound, positively charged carbenium‐ion fragment and a forming, neutral but electron‐rich alkene fragment. During the separation process, a significant charge shift occurs as the transition state decomposes into the alkene product and the surface‐bound carbenium ion. When EFAl species (highly polarizable Al oxo/hydroxo species) are present near SBAS, they can further interact with the positively charged transition state and contribute to the stabilization of the two separating fragments through additional electrostatic interactions. This interaction shifts the transition state toward a later, more dissociative configuration in which the two product‐like fragments (e.g., propene and the ethyl carbenium ion) are further separated [50]. This shift increases the rotational and vibrational degrees of the freedom of the fragments, and consequently the transition entropy is expected to increase.

By definition, if the carbenium ion constitutes the transition state, formation of the structure depicted in Scheme 3 must be associated with a lower energy than that of the carbenium transition state. However, the physical elongation of the *β*‐bond and shifting the charge of the carbenium ion to fragment must increase the energy, making the structure in Scheme 3 the transition state. Of course, this does not change the transition energy of pentene monomolecular cracking as 167 kJ·mol^−1^ (Scheme 2), but it places the structure in Scheme 3 at this energy rather than the most stable carbenium ion.

## 3 Conclusion

This study provides a quantitative and mechanistic framework for alkene cracking in zeolites, using linear pentenes as model substrates. Cracking is initiated from π‐bonded alkenes and proceeds via carbenium‐ion‐like transition states, whereas alkoxide species do not play a kinetically relevant role under cracking conditions.

Monomolecular cracking, probed through ethene formation, exhibits a reaction order close to one and an intrinsic activation enthalpy of Δ*H* ^ǂ^°_int._ = 167 kJ·mol^−1^. Comparison with *n*‐pentane cracking reveals that pentene cracking proceeds with a 26 kJ·mol^−1^ lower intrinsic enthalpic barrier, while intrinsic activation entropies are comparable (−3 and 3 J·mol^−1^·K^−1^, respectively). The markedly higher cracking activity of alkenes relative to alkanes on H‐ZSM‐5 (*k*_pentene_/*k*_pentane_ ≈ 28 at 773 K) is therefore governed primarily by enthalpic stabilization of the carbenium‐ion transition state.

Beyond monomolecular cracking, the pentene system enables differentiation between pathways involving carbenium ions of different stability. Two principal routes to butene formation were identified: (i) dimerization cracking (type B_2_), proceeding via tertiary‐to‐secondary carbenium ions, and (ii) monomolecular cracking (type D_2_’/E_2_) involving formation of a CH_3_^+^ fragment. At low temperatures (703–733 K), dimerization cracking dominates, characterized by a reaction order near two and a low intrinsic activation enthalpy (Δ*H* ^ǂ^°_int._ = 64 kJ·mol^−1^). At higher temperatures (813–843 K), this pathway becomes entropically disfavored and butene formation is governed instead by monomolecular cracking with a substantially higher intrinsic enthalpic barrier (Δ*H* ^ǂ^°_int._ = 184 kJ·mol^−1^).

Alkene cracking is further shown to be highly sensitive to the local environment surrounding the active site. Narrower pore systems suppress the reaction rate by imposing enthalpic penalties on bulky carbenium‐ion‐like transition states, as reflected by enthalpy‐entropy compensation across different zeolite frameworks. In contrast, modification of the active site vicinity by EFAl does not primarily change the intrinsic enthalpic barrier but shifts the transition state toward a later, more dissociative configuration resulting in a substantially less negative entropic barrier and higher rates. This shift reflects the higher configurational freedom of the transition state within the modified environment.

Overall, alkene cracking in zeolites emerges as a process governed by a balance of intrinsic chemical reactivity and environment‐dependent enthalpic and entropic contributions. Pore topology and active‐site modification jointly determine whether rates are limited by steric stabilization of the transition state or by configurational freedom along the reaction coordinate. These insights provide a mechanistic basis for tailoring zeolite catalysts and reaction conditions to manipulate the product spectrum and establish a foundation for understanding more complex reaction environments involving mixing alkane and alkene feeds.

## Author Contributions

**Ruixue Zhao**: writing – review and editing, conceptualization, investigation. **Stefan Schallmoser**: writing – original draft, investigation. **Gary L. Haller**: conceptualization, writing – review, and editing. **Maricruz Sanchez‐Sanchez**: conceptualization, writing – original draft. **Johannes A. Lercher**: conceptualization, writing – review and editing, supervision.

## Conflicts of Interest

The authors declare no conflicts of interest.

## Supporting information




**Supporting File**: anie72422‐sup‐0001‐SuppMat.docx.

## Data Availability

The data that support the findings of this study are available from the corresponding author upon reasonable request.
