# Supplementary material for: Consequences of Medium‐Pore Zeolite Constraints for Alkene Cracking—The Case of n‐Pentene
Source: Angew Chem Int Ed Engl. 2026 Apr 30;65(25):e4217298. doi: 10.1002/anie.4217298 (PMC13266936; doi:10.1002/anie.4217298)
Supplement: Supplementary file 1 — Supporting File: anie72422‐sup‐0001‐SuppMat.docx. [file ANIE-65-e4217298-s001.docx]

**Supporting Information**

Consequences of medium-pore zeolite constraints for alkene cracking—the case of *n*-pentene

Ruixue Zhao^a,*^, Stefan Schallmoser^a^, Gary L. Haller^b^, Maricruz Sanchez-Sanchez^a,c^, Johannes A. Lercher^a,*^

^a^ Department of Chemistry, TUM School of Natural Sciences, Catalysis Research Center, Technical University Munich, 85747 Garching, Germany

^b^ Department of Chemical and Environmental Engineering, Yale University, New Haven, CT 06520, United States

^c^ Institute of Chemical Environmental and Bioscience Engineering, Vienna University of Technology, Vienna 1060, Austria

Content

[S.1 Experimental 4](#_Toc226551281)

[S.1.1 Catalysts information 4](#_Toc226551282)

[S.1.2 Zeolite synthesis 4](#_Toc226551283)

[S.1.3 EFAl-removal by AHFS-Treatment 4](#_Toc226551284)

[S.1.4 Static calcination 4](#_Toc226551285)

[S.1.5 X-Ray powder diffraction 4](#_Toc226551286)

[S.1.6 Elemental analysis 4](#_Toc226551287)

[S.1.7 N_2_-physisorption/desorption 4](#_Toc226551288)

[S.1.8 Characterization of acid sites 5](#_Toc226551289)

[S.1.9 Kinetic measurements of pentene cracking 5](#_Toc226551290)

[S.2 Characterization of investigated catalysts 6](#_Toc226551291)

[S.2.1 Basic physiochemical properties of investigated catalysts 6](#_Toc226551292)

[Table S1. Physiochemical properties of investigated catalysts.^[6]^ Adapted with permission from Elsevier, © 2014 Elsevier Inc.. 6](#_Toc226551293)

[S.2.2 Identification and quantification of Al pairs 6](#_Toc226551294)

[Table S2. Ratios of Co, Na, and Al contents in MFI Zeolites with high Al content. 7](#_Toc226551295)

[S.2.3 Identification and quantification of different SBAS species 7](#_Toc226551296)

[Figure S1. Infrared spectra of OH stretching vibrations region of activated MFI zeolites (normalized to the overtone and combination region of ZSM-5 lattice vibrations between 2095 and 1735 cm^−1^). Adapted from ref.^[6]^ with permission from Elsevier, © 2014 Elsevier Inc.. 7](#_Toc226551297)

[Figure S2. Difference spectra of the OH stretching region for MFI-15-ST measured at 423 K: (a) after pyridine adsorption and subsequent heating to 723 K, including the deconvoluted components; (b) after sequential adsorption and outgassing of pyridine and ammonia at 423 K. Note the difference in scale on the y-axis. Adapted from ref.^[6]^ with permission from Elsevier, © 2014 Elsevier Inc.. 8](#_Toc226551298)

[Figure S3. 1D ^27^Al MAS NMR spectra for the MFI-15 series. Solid line represents the experimental spectrum, dashed line represents the fitting result, dark-colored area for Gaussian 1 (G1), light-colored area for Gaussian 2 (G2). Adapted from ref.^[6]^ with permission from Elsevier, © 2014 Elsevier Inc.. 8](#_Toc226551299)

[Table S3. Concentrations of overall SBAS, iso-SBAS, and EFAl-SBAS in the MFI-15 series.^[6]^ Adapted with permission from Elsevier, © 2014 Elsevier Inc.. 8](#_Toc226551300)

[S.3 Kinetic measurements 9](#_Toc226551301)

[S.3.1 *β*-scission cracking modes and the associated labels 9](#_Toc226551302)

[Table S4. *β*-scission cracking modes and the associated labels. 9](#_Toc226551303)

[S.3.2 Mechanistic considerations 9](#_Toc226551304)

[Scheme S1. Simplified scheme of main pathways in 1-pentene cracking on acidic catalysts, secondary reactions are not shown (Adapted from refs. ^[16, 18]^). 10](#_Toc226551305)

[Scheme S2. Cracking pathways of pentene isomers via direct *β*-scission. (Modes of *β*-scission and the associated labels can be found in Table S4. To clarify the reaction pathways, D_2_ denotes the 2°→1° cracking route that does not generate a CH_3_^+^ fragment, whereas D_2_’ denotes the 2°→1° cracking route that generate a CH_3_^+^ fragment.) 10](#_Toc226551306)

[S.3.3 Weisz-Prater criterion for estimation of internal mass transport limitations 11](#_Toc226551307)

[Table S5. Measured highest rate (*r*_meas._) at selected temperatures and the corresponding Weisz–Prater criterion (CWP). 11](#_Toc226551308)

[S.3.4 Reaction performance 11](#_Toc226551309)

[Figure S4. Consumption rate of 1-pentene at 763 K as function of TOS (shown for sample MFI-90). 12](#_Toc226551310)

[Figure S5. Relative selectivity to branched and linear pentene isomers as function of conversion (T = 763 K). Dash lines indicate selectivities in the equilibrium. 12](#_Toc226551311)

[Figure S6. Relative selectivity to butene isomers as function of conversion (T = 763 K). 12](#_Toc226551312)

[Figure S7. Arrhenius correlation for overall cracking rate of *n*-pentane in MFI-90. 13](#_Toc226551313)

[Figure S8. The rate for ethene (a) and butene (b–e) formation as a function of pentene partial pressure with a total flow of 400 mL·min^−1^ (N_2_ as carrier gas) at 713–733 K. *m*_cat._=5–15 mg (250–315 µm), charged with SiC dilution (250–315 µm fraction, 295–285 mg). Pentene conversion was below 5%. 13](#_Toc226551314)

[Figure S9. Deactivation behavior of the MFI-15 series, H-TON and H-FER (T=763 K) shown as function of TOS. Deactivation index represents the rate normalized by the initial activity. Initial conversion was about 5 % for all five samples. 13](#_Toc226551315)

[Figure S10. The XRD patterns of fresh and used catalyst. 14](#_Toc226551316)

[S.3.5 Kinetic analysis 14](#_Toc226551317)

[References 15](#_Toc226551318)

# S.1 Experimental

## S.1.1 Catalysts information

Five commercial H-ZSM-5 samples provided by Zeolyst International (MFI-15 = CBV3024E, MFI-25 = CBV5524G and MFI-40 = CBV8014) and Clariant AG (MFI-60 and MFI-90) were used. Two additional H-ZSM-5 catalysts were synthesized to extend the investigation to higher Si/Al ratios. Depending on their composition, the samples were designated as MFI-X, with “X” relating to their overall Si/Al ratio (atom/atom). NH_4_^+^-FER (FER) was obtained from Zeolyst (CP 914C). NH_4_^+^-ZSM-22 (TON) was received from Dr. M. Derewinski of the Polish Academy of Science. The sample was calcined for 5 h in a stream of synthetic air (100 mL·min^-1^, heating rate 10 K·min^-1^, 823 K) to yield the H-form.

## S.1.2 Zeolite synthesis

The samples with high Si/Al ratios (MFI-240 and MFI-470) were prepared by hydrothermal synthesis according to the following procedure. For H-MFI-240, 0.42 g (1.1 mmol) of Al(NO_3_)_3_∙9H_2_O (purity ≥ 98%, Sigma-Aldrich) were dissolved in 30 ml of deionized water and slowly added to a solution of 3.0 g (75 mmol) NaOH (purity ≥ 98%, Fluka-Analytical) dissolved in 30 ml of deionized water. The corresponding amount of Al(NO_3_)_3_∙9H2O for H-MFI-470 was 0.21 g. The resulting solution was clear (solution A). Then, another solution, containing 14.8 g (55 mmol) tetrapropyl ammonium bromide (TPABr, purity ≥ 98%, Sigma-Aldrich) and 1.51 g (38 mmol) NaOH dissolved in 70 ml of deionized water, was added to solution A. To the resulting, clear solution, 20 g of a fumed silica nano-powder (Cabosil M-5, Supelco-Analytical) were added gradually under vigorous stirring. The resulting viscous mixture was then stirred for 20 h at room temperature to yield an opaque gel. The subsequent hydrothermal synthesis was carried out in an autoclave with a PTFE liner for 24 h at 423 K. The resulting material was separated from the mother liquor, washed twice with deionized water, and calcined for 5 h in a stream of synthetic air (100 mL·min^-1^, heating rate 10 K·min^-1^, 823 K) to oxidatively remove the organic template. The crystallographic purity of the Na-ZSM-5 samples was verified by X-ray diffraction (not shown).

Subsequently, the as-synthesized samples were ion-exchanged three times at 353 K for 2 h, each time using 150 ml of a 1 M NH_4_NO_3_ solution (corresponding to 25 ml per 1 g of sample), to produce NH_4_-ZSM-5 zeolites. The resulting samples were dried at 393 K for 2 h and calcined for 5 h in a stream of synthetic air (100 mL·min^-1^, heating rate 10 K·min^-1^, 823 K) to yield the catalytically active H-forms.

## S.1.3 EFAl-removal by AHFS-Treatment

NH_4_-ZSM-5 (approx. 2 g of sample per 80 ml of solution) was added to a solution of (NH_4_)_2_SiF_6_ (AHFS) at 353 K and then stirred vigorously for 5 h. The synthesis was done in a PTFE-liner with a volume of 100 ml. The solution contained a 4-fold excess of AHFS with respect to the Al-content of the sample. This was 1.42 g (8.0 mmol) AHFS for 1.92 g of MFI-15 (2.9 wt-% Al corresponding to 2 mmol) and 0.88 g (5.0 mmol) AHFS for 1.92 g of MFI25 (1.7 wt-% Al corresponding to 1.3 mmol). After the AHFS treatment, the samples were washed six times in hot deionized water (353 K) and calcined for 5 h in a stream of synthetic air (100 mL·min^-1^, heating rate 10 K·min^-1^, 823 K). The samples modified by AHFS treatment were designated as MFI-XAHFS, where “*X*” stands for the overall Si/Al ratio (atom/atom) as determined by atomic absorption spectroscopy.

## S.1.4 Static calcination

The H-form of MFI-15 was placed as shallow bed (approx. 2 g, layer thickness ≈ 2-3 mm) into a quartz boat and was then heated under static atmospheric conditions (no flow of gas, ambient air present) with a temperature ramp of 10 K·min^-1^ to 823 K and kept there for 30 minutes, before it was allowed to cool to room temperature. This sample was designated as MFI-15-ST.

## S.1.5 X-Ray powder diffraction

X-Ray powder diffraction (XRD) determined the crystal structure for all samples. The Philips X’Pert Pro instrument used Cu-K_α_ radiation of 0.154056 nm (45 kV and 40 mA) and a rotating powder sample holder applying a step size of 0.019° /s in the 2θ range of 5° to 70°.

## S.1.6 Elemental analysis

The elemental composition of the samples was determined by atomic absorption spectroscopy in a Unicam M Series Flame-AAS equipped with an FS 95 autosampler and a GF 95 graphite furnace.

## S.1.7 N_2_-physisorption/desorption

Specific surface area and porosity were determined from N_2_ adsorption/desorption isotherms recorded on an automated PMI Sorptomatic 1990 instrument at liquid N_2_ temperature (77 K). The samples were outgassed in vacuum (*p* = 1×10^–3^ mbar) for 2 h at 723 K prior to adsorption.

## S.1.8 Characterization of acid sites

In order to determine the acid site concentration, temperature programmed desorption experiments of adsorbed ammonia (NH₃-TPD) and IR spectroscopy of adsorbed pyridine were used following the procedure described earlier.^[^[^1^](#_ENREF_1)^,^ [^2^](#_ENREF_2)^]^ Briefly, in a typical experiment of NH₃-TPD, approximately 50 mg of sample (500–710 μm) was first activated under vacuum (≤ 10⁻³ mbar) at 723 K for 1 h with a heating rate of 10 K·min⁻¹. After cooling to 373 K, NH₃ was introduced (1 mbar) and allowed to adsorb for 1 h. The excess NH₃ was then removed by evacuation for 2 h. Subsequently, the temperature was ramped to 1043 K at 7 K·min⁻¹, and desorbed NH₃ was continuously monitored by mass spectrometry (m/z = 16). The total acidity was quantified by integrating the desorption peaks and comparing the signal with that of a reference zeolite (H-ZSM-5, SiO₂/Al₂O₃ = 90, 0.360 mmol H⁺·g⁻¹, Süd-Chemie AG). The concentrations of Brønsted (BAS) and Lewis acid sites (LAS) were determined by pyridine adsorption at 423 K, followed by evacuation for 1 h to remove physisorbed species. To quantify strong Brønsted (SBAS) and strong Lewis acid sites (SLAS), the samples were subsequently heated to 723 K at a rate of 10 K·min⁻¹ and held for 30 min. After cooling to 423 K, an additional IR spectrum was collected for analysis. The pyridine adsorption sites that remained after heating to 723 K were defined as SBAS and SLAS. The difference spectra obtained after heating to 723 K were deconvoluted using two Gaussian components centered at 3656 and 3605 cm⁻¹, corresponding to distinct hydroxyl species on the MFI-15 and MFI-15-ST samples.

## S.1.9 Kinetic measurements of pentene cracking

Cracking of pentenes at atmospheric pressure was studied on H-ZSM-5 (250-315 µm pellets, 5–150 mg) charged with SiC dilution (250–315 µm fraction, 295–150 mg) into a quartz tube (ø = 6 mm). The temperature was measured at the wall of the quartz tube. Prior to the kinetic measurements, the catalysts were activated in synthetic air (30 mL·min^-1^) at 843 K for 2 h. After flushing for 30 min with N_2_, evaporated 1-pentene (>99.3% purity) was introduced in a mixture with N_2_ (total flow 100–400 mL·min^-1^, 1-pentene concentration 0.05–12 vol.%) at atmospheric pressure. The product stream was analyzed online with a gas chromatograph (Agilent 7890A) with DB-1, HayeSepQ and Molecular Sieve 13X columns separating the product stream. The gas chromatograph was equipped with FID and TCD detectors for analysis of all products including non-carbonaceous compounds. In the first step, deactivation was investigated for MFI-90. After 6 h time on stream (TOS), the pentene consumption rate decreased by 15% of its initial value, and deactivation was found to be in quasi steady state (**Figure S1**, less than 5% activity loss in the following 10 h TOS). Therefore, the catalyst was allowed to run for 6 h prior to all measurements determining kinetic parameters.

Isomerization among the six pentene isomers is expected to be faster than cracking, and, therefore, it is assumed that the ratio between the six isomers is close to the thermodynamic equilibrium. The pentene isomer distribution is a function of both partial pressure of 1-pentene and space velocities, but above 560 K, all pentene isomers form an “equilibrium pentene pool”.^[^[^3^](#_ENREF_3)^]^ Recently, Chen et al. reported that equilibration of C_4_-C_6_ alkene isomers is not always achieved in ZSM-5 at alkene cracking temperatures, due to the steric constrains favoring non-branched isomers.^[^[^4^](#_ENREF_4)^]^ However, analysis of effluent gases at 763 K at different conversions showed that, for conversions larger than 5%, linear and branched pentene isomers were equilibrated (**Figure S2**). For conversions below 5%, linear isomers are slightly favored. In the present study, all pentene isomers are treated as one pentene pool for further kinetic analysis. Conversion *X* and selectivity *S* are expressed according to **Eq. S1** and **Eq.S2**:

$X=1-\frac{\sum C_{pentene isomers}}{C_{initial, 1­pentene}}$ (**Equation S1**)

$S_{i}=1-\frac{n_{i}}{\sum n_{j}}\times100\%$ (**Equation S2**)

where *n_i_* is the moles of product *i* formed, and ∑𝑛_𝑗_ is the total moles of all products formed from pentene conversion.

For the highly active samples (MFI-15, MFI-25 and MFI-15-ST) dilution with silica (Aerosil 300) was necessary in order to assure a homogeneous distribution in the reactor bed. For doing so, 250 mg of silica were mixed carefully in a mortar with 25 mg of zeolite. The resulting powder was used for pelletizing. Blank tests showed that pure silica was inactive for 1-pentene cracking at 763 K. Due to the deactivation, temperature dependence of the rate constants was determined for the MFI-15 series by reactivation at 823 K in a flow of synthetic air (100 mL·min^-1^, heating rate 10 K·min^-1^, 1h) before each temperature step. Rates for each temperature were then extrapolated to initial TOS. These extrapolated values were used for the comparison and the calculation of activation energies using Eyring equation.

Blank reactor experiments were performed to exclude contributions of gas phase reactions. Absence of internal mass transport limitations was confirmed by evaluating the Weisz-Prater criterion (**Supporting Information S.3.3**). ^[^[^5^](#_ENREF_5)^]^ The apparent enthalpies and entropies for different reaction pathways are determined using the Eyring equation (**Supporting Information S.3.5**).

# S.2 Characterization of investigated catalysts

## S.2.1 Basic physiochemical properties of investigated catalysts

Table S1. Physiochemical properties of investigated catalysts.^[^[^6^](#_ENREF_6)^]^ Adapted with permission from Elsevier, © 2014 Elsevier Inc..

| Name^a^ | S_BET_ | Si:Al^a^ | BAS^b^ | SBAS^b^ | LAS^b^ | SLAS^b^ | BAS^c^ |
| --- | --- | --- | --- | --- | --- | --- | --- |
|  | [m^2^ g^-1^] | - | [µmol g^-1^] | | | | |
| MFI-15 | 405 | 15 | 843 | 705 | 213 | 168 | 820 |
| MFI-15-AHFS | 409 | 27 | 717 | 669 | 51 | 36 | 691 |
| MFI-15-ST | 415 | 17 | 444 | 376 | 234 | 167 | 398 |
| MFI-25 | 425 | 25 | 564 | 496 | 137 | 94 | 538 |
| MFI-25-AHFS | 446 | 26 | 425 | 396 | 31 | 21 | 449 |
| MFI-40 | 425 | 39 | 374 | 324 | 78 | 66 | 353 |
| MFI-60 | 512 | 58 | 286 | 257 | 59 | 39 | 238 |
| MFI-90 | 454 | 88 | 141 | 135 | 33 | 29 | 158 |
| MFI-240 | 431 | 240 | 62 | 57 | 11 | 10 | 68 |
| MFI-470 | 400 | 470 | 30 | 29 | 7 | 3 | 36 |
| H-FER | 397 | 25 | - | 200^d^ | - | - | 560 |
| H-TON | 360 | 32 | 327 | 291 | 36 | 30 | 349 |

^a^ Molar ratio of Si and Al determined with AAS;

^b^ Determined by IR spectroscopy of adsorbed pyridine;

^c^ Determined by NH_3_ TPD;

^d^ From Ref^[^[^2^](#_ENREF_2)^]^.

## S.2.2 Identification and quantification of Al pairs

The presence of Al pairs may influence the reaction rate of olefin cracking. Such Al pairs are predominantly found in samples with higher Al content, such as MFI-15, MFI-25 and MFI-40. To quantify the amount of framework Al associated with exchangeable Brønsted protons and Al pairs, Na^+^ form (Na-MFI-X) and Na^+^Co^2+^ form (Na-Co-MFI-X) zeolites were prepared from corresponding H^+^ form samples following the method reported by A. Janda and A. T. Bell.**^[^**[**^7^**](#_ENREF_7)**^]^** Briefly, Na-MFI-X zeolites were obtained by treating 1–2 g of the H^+^ form MFI-X with 100 mL of 1 M NaNO₃ at 353 K under stirring for 6 h, followed by filtration and washing with deionized water. The ion exchange was repeated three times. The dried samples were then heated in synthetic air (100 mL min⁻¹) to 393 K (1 K min⁻¹, 2 h), then to 773 K (2 K min⁻¹, 4 h), and cooled to ambient temperature. Na-Co-MFI-X samples were prepared by exchanging 1–2 g of Na-MFI-X zeolites with 0.05 M Co(NO₃)₂ solution (50 mL g⁻¹) for 24 h at room temperature. The exchange and subsequent washing/filtration steps were each repeated three times to ensure complete ion exchange and removal of free cobalt oxides. The content of Na, Co and Al were determined with atomic absorption spectroscopy (AAS). As trace amounts of metal species can influence the alkene isomerization,^[^[^8^](#_ENREF_8)^]^ these ion-exchanged samples were used solely for characterization and were not employed in catalytic experiments, which were performed on H-form zeolites.

As shown in **Table S2**, the Na/Al ratios of Na-MFI-X samples represent the amount of exchangeable BAS protons, that is, the framework Al content in the MFI-X zeolites. Both MFI-15 and MFI-25 contain a noticeable fraction of extra-framework Al (EFAl), as indicated by Na/Al < 1. In contrast, the Na/Al ratio on MFI-40 is close to unity (0.980), suggesting a minimal presence of EFAl in this sample. These observations are consistent with the BAS and LAS concentrations determined by pyridine adsorption (**Table S1**).

The lower overall exchange degree in Na-Co-MFI-X samples, reflected by smaller (2Co + Na)/Al ratios compared to Na/Al, indicates partial replacement of Na**^+^** by H**^+^** during the treatment, consistent with the findings reported by A. Janda and A. T. Bell.**^[^**[**^7^**](#_ENREF_7)**^]^** The 2Co/Al ratio represents the fraction of paired Al sites, as Co**^2+^** ions can only counter the charge of Al pairs. **^[^**[**^7^**](#_ENREF_7)**^]^** The three high-Al-content samples (MFI-15, MFI-25, and MFI-40) contain a noticeable fraction of Al pairs. However, MFI-40 exhibits an identical rate normalized to the number SBAS compared with the lower-Al-content samples (**Figure 3**). Moreover, selective removal of EFAl species in MFI-15 and MFI-25 also results in samples showing comparable pentene cracking rate per SBAS. Therefore, it can be concluded that Al pairs do not exert significant influence on the pentene cracking rate under the studied reaction conditions and will not be further discussed.

Table S2. Ratios of Co, Na, and Al contents in MFI Zeolites with high Al content.

| Name | Na/Al^a^ | (2Co + Na)/Al^b^ | 2Co/Al^b^ |
| --- | --- | --- | --- |
| MFI-15 | 0.733 | 0.581 | 0.307 |
| MFI-25 | 0.786 | 0.592 | 0.235 |
| MFI-40 | 0.980 | 0.649 | 0.269 |

Determined with AAS; ^a^ measured on Na^+^ form zeolites (Na-MFI-X); ^b^ measured on Na^+^Co^2+^ form zeolites (Na-Co-MFI-X).

## S.2.3 Identification and quantification of different SBAS species

The concentration of different SBAS species (iso-SBAS and EFAl-SBAS) were investigated in detail in our previous work combining IR spectroscopy and **^27^**Al NMR.^[^[^6^](#_ENREF_6)^,^ [^9^](#_ENREF_9)^]^ A brief discussion is provided here to clarify the quantification of overall SBAS, iso-SBAS and EFAl-SBAS using MFI-15 series as an example.

The nature of extra-framework alumina (EFAl) species was investigated for the parent MFI-15 zeolite and its modified derivatives obtained by static calcination (MFI-15-ST) and AHFS treatment (MFI-15-AHFS). Static calcination led to mild dealumination (Si/Al = 17) and an approximately 50% decrease in strong Brønsted acid sites (SBAS), while strong Lewis acid sites (SLAS) remained nearly constant (**Table S1**). In contrast, AHFS treatment caused more extensive dealumination (Si/Al = 27), removing approximately 80% of SLAS but only around 5% of SBAS (**Table S1**). IR spectra revealed a clear correlation between the decrease in the bridging hydroxyl band at 3610 cm⁻¹ and the appearance of an Al–OH vibration at 3656 cm⁻¹, suggesting partial transformation of framework tetrahedral Al³⁺ into extra-framework Al(OH)ₓ species. (**Figure S1**)

The interaction of these Al–OH groups with probe molecules provided further insight into their acidic nature. Pyridine adsorption caused the disappearance of both the 3605 cm⁻¹ and 3656 cm⁻¹ bands, whereas ammonia affected only a small fraction of the Al–OH groups, indicating that these sites are not strongly Brønsted acidic but can engage in lateral interactions with pyridinium ions located near SBAS. Deconvolution of the OH stretching region using Gaussian functions centered at 3656 cm⁻¹ and 3605 cm⁻¹ (**Figure S2**) enabled quantification of Brønsted sites with and without adjacent Al–OH groups (denoted as EFAl-SBAS and iso-SBAS, respectively). Complementary ²⁷Al MAS NMR analyses (**Figure S3**) identified a distorted tetrahedral Al species (55.1 ± 0.5 ppm, Gaussian 1) that coexists with regular framework Al (54.6 ± 0.3 ppm, Gaussian 2). The growth of this broad signal at 55 ppm, together with the 3656 cm⁻¹ IR band, confirms that the extra-framework Al–OH species originate from slightly distorted tetrahedral Al(OH)ₓ clusters located within the zeolite micropores, in proximity to strong Brønsted acid sites. Therefore, the concentrations of EFAl–SBAS and iso–SBAS were determined according to our previous study**^[^**[**^6^**](#_ENREF_6)**^]^** and are summarized in **Table S**3.

Figure S1. Infrared spectra of OH stretching vibrations region of activated MFI zeolites (normalized to the overtone and combination region of ZSM-5 lattice vibrations between 2095 and 1735 cm^−1^). Adapted from ref.^[^[^6^](#_ENREF_6)^]^ with permission from Elsevier, © 2014 Elsevier Inc..

Figure S2. Difference spectra of the OH stretching region for MFI-15-ST measured at 423 K: (a) after pyridine adsorption and subsequent heating to 723 K, including the deconvoluted components; (b) after sequential adsorption and outgassing of pyridine and ammonia at 423 K. Note the difference in scale on the y-axis. Adapted from ref.^[^[^6^](#_ENREF_6)^]^ with permission from Elsevier, © 2014 Elsevier Inc..

Figure S3. 1D ^27^Al MAS NMR spectra for the MFI-15 series. Solid line represents the experimental spectrum, dashed line represents the fitting result, dark-colored area for Gaussian 1 (G1), light-colored area for Gaussian 2 (G2). Adapted from ref.^[^[^6^](#_ENREF_6)^]^ with permission from Elsevier, © 2014 Elsevier Inc..

Table S3. Concentrations of overall SBAS, iso-SBAS, and EFAl-SBAS in the MFI-15 series.^[^[^6^](#_ENREF_6)^]^ Adapted with permission from Elsevier, © 2014 Elsevier Inc..

| Name | Area G1 | Area G2 | Overall-SBAS | EFAl-SBAS | Iso-SBAS |
| --- | --- | --- | --- | --- | --- |
|  | [3656 cm^-1^] (a.u.) | [3605 cm^-1^] (a.u.) | [µmol g^-1^] | [µmol g^-1^] | [µmol g^-1^] |
| MFI-15 | 0.43 | 8.90 | 705 | 34 | 671 |
| MFI-15-AHFS | Not visible | n.d | 669 | 0 | 669 |
| MFI-15-ST | 1.41 | 6.09 | 376 | 87 | 289 |

Ammonium hexafluorosilicate (AHFS) was employed as a mild and selective dealumination agent to remove extra-framework aluminum (EFAl) species while preserving the integrity of the zeolite framework. Previous studies have shown that, under controlled conditions, AHFS achieves more selective EFAL extraction with minimal framework degradation compared to conventional acid or chelating treatments.^[^[^10-13^](#_ENREF_10)^]^ We have demonstrated in our previous studies that, under the conditions applied here, AHFS treatment removes over 80% of the EFAl species (LAS/SLAS) while causing only a slight decrease in the framework aluminum (BAS/SBAS).^[^[^6^](#_ENREF_6)^,^ [^14^](#_ENREF_14)^,^ [^15^](#_ENREF_15)^]^ For the MFI-15 series examined in this study (**Table S1** and **S3**, **Figure S1** and **S3**), AHFS treatment markedly increased the Si/Al ratio from 15 to 27 by selectively remove EFAl species, as evidenced by an approximately 80% decrease in the concentration of SLAS, the disappearance of the EFAl-OH vibration band in the IR spectra, and the retention of approximately 95% of the SBAS. Under the mild AHFS treatment conditions applied here, silicon redeposition is negligible and does not affect the catalytic properties of the Brønsted acid sites. Our previous work has shown that chemically grafted silicon species can lower the activation energy for pentane cracking and enhance the reaction rate,^[^[^15^](#_ENREF_15)^]^ however, no such effect is observed for the AHFS-treated samples. The identical turnover frequencies (TOFs) of AHFS-treated and untreated samples with low Al content (**Figure 3**) confirm that silicon deposition plays no significant role under the present conditions, and the observed changes in activity can therefore be attributed primarily to the removal of EFAL species.

# S.3 Kinetic measurements

## S.3.1 *β*-scission cracking modes and the associated labels

Table S4. *β*-scission cracking modes and the associated labels.

| Cracking modes | Labels |  |
| --- | --- | --- |
| 3°→3° | A |  |
| 2°→3° | B_1_ | B |
| 3°→2° | B_2_ |  |
| 2°→2° | C |  |
| 1°→2° | D_1_ | D |
| 2°→1° | D_2_, D_2_’ * |  |
| 1°→3° | E_1_ | E |
| 3°→1° | E_2_ |  |
| 1°→1° | F |  |

1°, 2° and 3° represent primary, secondary and tertiary carbenium ion;

* To clarify the reaction pathways, D_2_ denotes the 2°→1° cracking route that does not generate a CH_3_^+^ fragment, whereas D_2_’ denotes the 2°→1° cracking route that generate a CH_3_^+^ fragment.

## S.3.2 Mechanistic considerations

Mechanistically, two principal pathways coexist in the cracking of linear pentenes: **Rxn. 1,** the monomolecular cracking pathway; **Rxn. 2**, dimerization/oligomerization followed by cracking.^[^[^16^](#_ENREF_16)^,^ [^17^](#_ENREF_17)^]^

C_5_H_10_ → C_2_H_4_ + C_3_H_6_ (**Reaction 1**)

2 C_5_H_10_ → C_10_H_20_ → C_4_H_8_ + C_6_H_12_ (**Reaction 2**)

**Scheme S1** illustrates further possible pathways and side reactions commonly observed for pentene cracking on acid catalysts/zeolites. Especially for higher conversions, also the primary products (ethene, propene, butenes) are subject to further alkylation and subsequent cracking of the formed larger alkenes. ^[^[^16^](#_ENREF_16)^,^ [^17^](#_ENREF_17)^]^

Scheme S1. Simplified scheme of main pathways in 1-pentene cracking on acidic catalysts, secondary reactions are not shown (Adapted from refs. ^[^[^16^](#_ENREF_16)^,^ [^18^](#_ENREF_18)^]^).

Scheme S2. Cracking pathways of pentene isomers via direct *β*-scission. (Modes of *β*-scission and the associated labels can be found in Table S4. To clarify the reaction pathways, D_2_ denotes the 2°→1° cracking route that does not generate a CH_3_^+^ fragment, whereas D_2_’ denotes the 2°→1° cracking route that generate a CH_3_^+^ fragment.)

Alkenes form primary (1°), secondary (2°) and tertiary (3°) carbenium ions, differing in relative stabilities. Buchanan et al.^[^[^17^](#_ENREF_17)^]^ introduced a refined nomenclature of Weitkamp et al.^[^[^19^](#_ENREF_19)^]^ for the various types of *β*-scission that may occur in alkene cracking, reflecting the differences of carbenium ion reactivities throughout cracking. According to this nomenclature, the possible pathways of monomolecular cracking of pentene (**Rxn. 1**) are enumerated in **Scheme S2**. **Rxn. 1** includes cracking type of D_1_ (1°→2°), D_2_ (2°→1°), D_2_’ (D**_2_** cracking pathways generating CH**_3_^+^** fragment), E_2_ (3°→1°) and F (1°→1°). As the generated C_5_ primary carbenium ion intermediates of D_1_, E_2_ and F types are thermodynamic unfavored compared to the secondary carbenium ion intermediate of D_2_, and the CH**_3_^+^** fragment derived from D_2_’ is likewise unfavorable, D_2_ is expected to dominate **Rxn. 1**. Thus, **Rxn. 1** can be classified as type D_2_, as will be discussed below. For **Rxn. 2** (dimerization cracking/bimolecular cracking) a large number of C_10_ alkenes that are formed from six different pentene isomers need to be considered. Nevertheless, the dominant transitions will involve cracking from a tertiary carbenium ion to a secondary, assuming that dimethyl substitution is improbable due to steric restrictions within the zeolite. Therefore, we classify **Rxn. 2** as type B_2_ (modes of *β*-scission and the associated labels can be found in **Table S4**).

Please note that the observed reaction rates of the different pathways follow generally the order: B > D (**Scheme S2**, **Table S4**). Buchanan et al.^[^[^17^](#_ENREF_17)^]^ attributed this to a difference in population of the different carbenium ions (e.g., tertiary carbenium ions are more stable than secondary), without further analyzing the intrinsic energetic barriers of these pathways. Chen et al.^[^[^4^](#_ENREF_4)^]^ derived intrinsic energy barriers for different alkene cracking modes and found differences of up to 100 kJ·mol^-1^, depending on the postulated alkoxide species involved in the reaction. These markedly different intrinsic energy barriers are also predicted by theoretical calculations.^[^[^20^](#_ENREF_20)^]^

Parallel acid catalyzed isomerization of the alkenes must also be considered. Isomerization will have a faster rate than cracking; above 563 K, at the reaction temperatures used for cracking (703–843 K), both double bond and skeletal isomerization are expected to be quasi equilibrated.^[^[^3^](#_ENREF_3)^]^ We have experimentally verified that at the reaction temperature (703–843 K) and conversions (2–5 %) used in the present study the pentene isomers are quasi-equilibrated, with only a maximum deviation of 1.5 % in selectivities at the lowest conversions at 763 K (**Figure S5**, a similar trend is observed for the butene isomers as shown in **Figure S6)**. Therefore, the presence of six pentene isomers is included in mechanistic considerations (**Scheme S2**). While the skeletal pentene isomers are able to form (more stable) tertiary carbenium ions, cracking of these intermediates via *β*-scission is energetically hindered by the formation of CH_3_^+^ fragments (**Scheme S2**, type E_2_). In view of these considerations, we hypothesize that only linear isomers of pentene undergo direct monomolecular cracking via the D_2_ cracking mode. The other pathways, namely D_1_, D_2_̓ (D**_2_** cracking pathways generating CH**_3_^+^** fragment), E_2_ and F (**Scheme S2**), either forming a CH_3_^+^ fragment or going through a larger carbenium ion intermediate, are expected to contribute to the mechanism only at temperatures above 763 K.

## S.3.3 Weisz-Prater criterion for estimation of internal mass transport limitations

External mass transport limitations were evaluated by varying catalyst particle size (80–315 µm) and flow rate (100–400 mL·min^-1^). No dependence of the measured reaction rates on either parameter was observed, confirming that external diffusion is negligible under the conditions employed.

The presence of internal diffusion limitations was evaluated using the generalized Weisz–Prater criterion (*C_WP_*), which relates the observed reaction rate to the rate of diffusive transport inside a catalyst particle.^[^[^5^](#_ENREF_5)^]^ For a power-law rate expression *r*=*kC*^m^, the criterion is expressed as:

$$C_{WP}=\frac{m+1}{2}\frac{r_{meas.}\rho_{cat,vol}{L_{c}}^{2}}{c_{i,o}D_{\mathrm{eff}}}$$

where *r*_meas._ is the measured reaction rate per unit catalyst mass (mol·kg_cat_^−1^·s^−1^), *ρ*_cat,vol_ is the mass of solid per catalyst volume (kg·m^−3^), *L_c_* is the characteristic diffusion length of the particle (m), *c_i_*_,0_ is the bulk concentration of reactant *i* at the particle surface (mol·m^−3^), and *D*_eff_ is the effective diffusivity within the porous network (m²·s^−1^). The prefactor (m+1)/2 accounts for the influence of the apparent reaction order on the concentration profile inside the particle, and reduces to unity for a first-order reaction.

Values of *C_WP_* ≪ 0.3 indicate that internal diffusion limitations are negligible, whereas values approaching or exceeding unity suggest that pore diffusion significantly restricts the observed reaction rate.

In the present work, *L*_c_ is 1 µm taking the size of the primary zeolite crystals, a value of 1x10^−4^ cm² s^−1^ is employed for *D*_eff_, which is reported for 1-hexene.^[^[^21^](#_ENREF_21)^]^ The density of the catalyst is 1.8 × 10^3^ kg m^-3^ and the concentration of the reactant is 1.2 mol m^−3^. The calculated results of *C_WP_* for MFI-15 MFI-90, H-FER and H-TON are listed in **Table S1** (taking the highest rate for each catalyst). All the calculated *C_WP_* values are below 0.3 (**Table S5**), indicating the absence of internal transport limitations.

Table S5. Measured highest rate (*r*_meas._) at selected temperatures and the corresponding Weisz–Prater criterion (CWP).

| **Name** | ***T* [K]** | ***r*_meas._ [mol·g_cat_^-1^·s^-1^]** | ***C_WP_*** |
| --- | --- | --- | --- |
| **MFI-15** | 733 | 1.27×10^-4^ | 1.91×10^-2^ |
| **MFI-90** | 843 | 1.26×10^-5^ | 1.90×10^-3^ |
| **H-TON** | 843 | 1.09×10^-5^ | 1.63×10^-3^ |
| **H-FER** | 763 | 2.56×10^-7^ | 3.84×10^-5^ |

## S.3.4 Reaction performance

Catalysts with low Al content and clean BAS (i.e., without EFAl), such as MFI-90 and MFI-15-AHFS, display only limited deactivation. For these samples, the pentene consumption rate decreases by approximately 15% during the first 6 h and subsequently reaches a quasi–steady state, with less than 5% additional loss over the following 10 h (**Figure S4** and **S9**). Because these catalysts stabilize reliably, all kinetic measurements for these materials were collected after 6 h TOS to ensure steady-state performance.

In contrast, samples such as MFI-15, MFI-15-ST, TON, and FER exhibit continuous deactivation throughout the reaction and do not reach a steady state. For these catalysts, the measured reaction rates are inherently time-dependent and cannot be directly compared across temperatures. Therefore, prior to each temperature step, the catalyst was regenerated in synthetic air (823 K, 1 h, 10 K·min⁻¹), and the rate measured immediately after regeneration was extrapolated back to the initial TOS. These extrapolated initial rates were then used for all kinetic comparisons and for determining activation parameters using the Eyring equation.

This strategy ensures that intrinsic kinetic behavior is obtained for all samples, regardless of differences in stability or susceptibility to deactivation.

The possible contribution of mesopores or interparticle space formed during dealumination is expected to be negligible under the present conditions, as the reactions involve relatively small molecules and are carried out under differential conditions, where cracking predominantly occurs on Brønsted acid sites within the microporous framework.^[^[^22-26^](#_ENREF_22)^]^

Figure S4. Consumption rate of 1-pentene at 763 K as function of TOS (shown for sample MFI-90).

Figure S5. Relative selectivity to branched and linear pentene isomers as function of conversion (T = 763 K). Dash lines indicate selectivities in the equilibrium.

Figure S6. Relative selectivity to butene isomers as function of conversion (T = 763 K).

Figure S7. Arrhenius correlation for overall cracking rate of *n*-pentane in MFI-90.

Figure S8. The rate for ethene (a) and butene (b–e) formation as a function of pentene partial pressure with a total flow of 400 mL·min^−1^ (N_2_ as carrier gas) at 713–733 K. *m*_cat._=5–15 mg (250–315 µm), charged with SiC dilution (250–315 µm fraction, 295–285 mg). Pentene conversion was below 5%.

Figure S9. Deactivation behavior of the MFI-15 series, H-TON and H-FER (T=763 K) shown as function of TOS. Deactivation index represents the rate normalized by the initial activity. Initial conversion was about 5 % for all five samples.

Figure S10. The XRD patterns of fresh and used catalyst.

## S.3.5 Kinetic analysis

At the low conversion levels (2–5%) examined in this study, only the principal reactions outlined in the main manuscript (also listed below) are considered.

C_5_H_10_ → C_2_H_4_ + C_3_H_6_ (Reaction 1)

2 C_5_H_10_ → C_10_H_20_ → C_4_H_8_ + C_6_H_12_ (Reaction 2)

C_6_H_12_ → 2 C_3_H_6_ (Reaction 3)

Considering Rxn. 1 and Rxn. 2 as the primary pathways for the formation of the main products, the selectivities toward butenes and hexenes should vary in parallel, as should those toward propene and ethene. However, the product distributions (**Figure 1**) deviate from the stoichiometric expectations of Rxn. 1 and Rxn. 2 over a wide range of temperatures and conversions. For example, at 763 K and low conversions, the selectivity to propene (*S*_propene_) was 8.5% higher than that to ethene (*S*_ethene_), while the selectivity to butene (*S***_butene_**) exceeded that of hexene (*S*_hexene_) by about 5%. The ratio between these two differences was approximately 2:1. At low conversions, the selectivity differences between products originating from the same pathway, ethene/propene for monomolecular cracking (Rxn. 1) and butene/hexene for bimolecular or dimerization cracking (Rxn. 2), can be satisfactorily explained by the secondary cracking of hexene to propene (Rxn. 3). This reaction consumes hexene (but not butene) and increases the yield of propene without affecting ethene.

At the temperatures used for pentene cracking in this work (≥ 703 K), ethene dimerization to butenes can be neglected. Ethene oligomerization/dimerization on protonic (BAS) sites is typically observed only at much lower temperatures and elevated ethene partial pressures and in confined pore environments.^[^[^27-29^](#_ENREF_27)^]^ Under such conditions protonation and surface carbocation/ethoxy intermediates are sufficiently stabilized to give measurable butene formation. In contrast, experiments and mechanistic studies on H-ZSM-5/H-Beta show that increasing temperature shifts the reaction network toward monomolecular cracking, *β*-scission, isomerization and hydrogen-transfer pathways, while oligomerization rates (including dimerization) fall markedly, for example, ethylene conversion drops to only a few percent under high-T/low-P conditions.

Therefore, the rate of ethene formation is taken as a measure of the monomolecular cracking rate, whereas the rate of butene formation represents the bimolecular (dimerization) cracking rate. The possible contribution of other monomolecular cracking modes to butene formation, particularly at high temperatures, are discussed in the manuscript.

The main elemental steps for pentene cracking are:

physisorption: $C_{5}^{=}\underset{\Leftrightarrow}{K_{phys.}}{C_{5}^{=}}_{phys.}$ (*r*_1_)

*π*-complex formation: $C_{5}^{=}\underset{\Leftrightarrow}{K_{\pi}}{C_{5}^{=}}_{\pi}$ (*r*_2_)

monomolecular cracking: ${C_{5}^{=}}_{\pi}\underset{\to}{k_{mono.}}C_{2}^{=} + C_{3}^{=}$ (*r*_mono._)

bimolecular (dimerization) cracking: ${C_{5}^{=}}_{\pi}+ {C_{5}^{=}}_{phys.} \underset{\to}{k_{dimer.}}C_{4}^{=} + C_{6}^{=}$ (*r*_dimer_)

We tentatively propose that in bimolecular cracking, a π-complexed olefin is alkylated by an adjacent physisorbed pentene molecule. The subsequent cracking of the formed dimer—analogous to the monomer in monomolecular cracking—is presumed to be the rate-determining step, while all other steps are considered to be in quasi-equilibrium. **Consequently, the net forward rate**s for monomolecular and bimolecular cracking are governed solely **by** *r*_mono._ and *r*_dimer._, respectively**.**

Given the high reaction temperature (T > 703 K), adsorption of the pentene isomers is expected to occur within the linear regime of the adsorption isotherm, corresponding to low surface coverages for both physisorbed and *π*-complex species. Therefore, a Henry-type adsorption behavior can be reasonably assumed.

$\theta_{{c_{5}^{=}}_{phy.}}=K_{phys.} p_{c_{5}^{=}}$ (Equation S1)

$\theta_{{c_{5}^{=}}_{\pi}}=K_{\pi} p_{c_{5}^{=}}$ (Equation S2)

$r_{meas.,mono.}=r_{mono.}=k_{mono.}\theta_{{c_{5}^{=}}_{\pi}}=k_{mono.}K_{\pi}p_{c_{5}^{=}}=k_{meas.,mono.}p_{c_{5}^{=}}$ (Equation S3)

$r_{meas.,dimer.}=r_{dimer.}=k_{dimer.}\theta_{{c_{5}^{=}}_{\pi}}\theta_{{c_{5}^{=}}_{phy.}}=k_{dimer.}K_{\pi}K_{phys.}\left( p_{c_{5}^{=}} \right)^{2}=k_{meas.,dimer.}\left( p_{c_{5}^{=}} \right)^{2}$ (Equation S4)

where $\theta_{i}$ denotes the surface coverage of species ***i***, $K_{i}$ represents the equilibrium constant for step ***i***, $k_{i}$ represents the rate constant for step *i*, and $p_{c_{5}^{=}}$ denotes the partial pressure of pentene.

Therefore, the measured rate constant for monomolecular cracking and bimolecular cracking are:

$k_{meas.,mono.}=k_{mono.}K_{\pi}$ (Equation S5)

$k_{meas.,dimer.}=k_{dimer.}K_{\pi}K_{phys.}$ (Equation S6)

From Eyring equation based on transition state theory:

$k=\kappa\frac{k_{B}T}{h}e^{-\Delta G^{\ddagger}/RT}$ (Equation S7)

where $k$ is the rate constant, $\kappa$ is the transmission coefficient (assumed to be equal to one), $k_{B}$ is the Boltzmann constant (1.381×10^−^**^23^**J·K**^−1^**), ***T*** is the absolute temperature in Kelvin, ***h*** is the Planck constant (6.626×10^−34^J·s), $\Delta G^{\ddagger}$ is the Gibbs free energy of activation, ***R*** is the ideal gas constant (8.314 J·mol^−1^·K^−1^).

As the Gibbs free energy ($\Delta G$) is related to enthalpy ($\Delta H$), entropy ($\Delta S$), and temperature ($T$) by the equation:

$\Delta G=\Delta H-T\Delta S$ (Equation S8)

Therefore, the enthalpy and entropy can be determined by:

$\ln\frac{k}{T}=\frac{-\Delta H^{\ddagger}}{RT}+\ln\frac{k_{B}}{h}+\frac{-\Delta S^{\ddagger}}{R}$ (Equation S9)

Combine Equ. S9 with Equ. S5 and Equ. S6, the **intrinsic enthalpy** ($\Delta{H^{\ddagger^{\circ}}}_{int.}$) and entropy ($\Delta{S^{\ddagger^{\circ}}}_{int.}$) can be obtained:

monomolecular cracking

$\Delta{H^{\ddagger^{\circ}}}_{int.}=\Delta{H^{\ddagger^{\circ}}}_{meas.}-\Delta{H^{^{\circ}}}_{\pi}$ (Equation S10)

$\Delta{S^{\ddagger^{\circ}}}_{int.}=\Delta{S^{\ddagger^{\circ}}}_{meas.}-\Delta{S^{^{\circ}}}_{\pi}$ (Equation S11)

bimolecular cracking:

$\Delta{H^{\ddagger^{\circ}}}_{int.}=\Delta{H^{\ddagger^{\circ}}}_{meas.}-\Delta{H^{^{\circ}}}_{\pi}-\Delta{H^{^{\circ}}}_{phys.}$ (Equation S12)

$\Delta{S^{\ddagger^{\circ}}}_{int.}=\Delta{S^{\ddagger^{\circ}}}_{meas.}-\Delta{S^{^{\circ}}}_{\pi}-\Delta{S^{^{\circ}}}_{phys.}$ (Equation S13)

## References

[1] S. M. Maier, A. Jentys, J. A. Lercher, *The Journal of Physical Chemistry C* **2011**, *115*, 8005-8013.

[2] S. Schallmoser, G. L. Haller, M. Sanchez-Sanchez, J. A. Lercher, *Journal of the American Chemical Society* **2017**, *139*, 8646-8652.

[3] T. Mäurer, B. Kraushaar-Czarnetzki, *Journal of Catalysis* **1999**, *187*, 202-208.

[4] C.-J. Chen, S. Rangarajan, I. M. Hill, A. Bhan, *Acs Catalysis* **2014**, *4*, 2319-2327.

[5] P. Weisz, C. Prater, *Advances in catalysis* **1954**, *6*, 143-196.

[6] S. Schallmoser, T. Ikuno, M. Wagenhofer, R. Kolvenbach, G. Haller, M. Sanchez-Sanchez, J. Lercher, *Journal of Catalysis* **2014**, *316*, 93-102.

[7] A. Janda, A. T. Bell, *Journal of the American Chemical Society* **2013**, *135*, 19193-19207.

[8] S. Sanz-Navarro, M. Mon, A. Doménech-Carbó, R. Greco, J. Sánchez-Quesada, E. Espinós-Ferri, A. Leyva-Pérez, *Nature Communications* **2022**, *13*, 2831.

[9] J. Dědeček, D. Kaucký, B. Wichterlová, *Chemical Communications* **2001**, 970-971.

[10] C. S. Triantafillidis, A. G. Vlessidis, L. Nalbandian, N. P. Evmiridis, *Microporous and Mesoporous Materials* **2001**, *47*, 369-388.

[11] M. Abdolrahmani, K. Chen, J. L. White, *The Journal of Physical Chemistry C* **2018**, *122*, 15520-15528.

[12] A. Gola, B. Rebours, E. Milazzo, J. Lynch, E. Benazzi, S. Lacombe, L. Delevoye, C. Fernandez, *Microporous and Mesoporous Materials* **2000**, *40*, 73-83.

[13] H.-M. Kao, Y.-C. Chen, *The Journal of Physical Chemistry B* **2003**, *107*, 3367-3375.

[14] Y. Zhang, R. Zhao, M. Sanchez-Sanchez, G. L. Haller, J. Hu, R. Bermejo-Deval, Y. Liu, J. A. Lercher, *Journal of Catalysis* **2019**, *370*, 424-433.

[15] R. Zhao, R. Khare, Y. Zhang, M. Sanchez-Sanchez, R. Bermejo-Deval, Y. Liu, J. A. Lercher, *Nature Catalysis* **2023**, *6*, 68-79.

[16] O. Bortnovsky, P. Sazama, B. Wichterlova, *Applied Catalysis A: General* **2005**, *287*, 203-213.

[17] J. Buchanan, J. Santiesteban, W. Haag, *Journal of Catalysis* **1996**, *158*, 279-287.

[18] M. Höchtl, A. Jentys, H. Vinek, *Applied Catalysis A: General* **2001**, *207*, 397-405.

[19] J. Weitkamp, P. A. Jacobs, J. A. Martens, *Applied catalysis* **1983**, *8*, 123-141.

[20] M. N. Mazar, S. Al-Hashimi, M. Cococcioni, A. Bhan, *The Journal of Physical Chemistry C* **2013**, *117*, 23609-23620.

[21] W. O. Haag, R. M. Lago, P. B. Weisz, *Faraday Discussions of the Chemical Society* **1981**, *72*, 317-330.

[22] P. Peng, X.-H. Gao, Z.-F. Yan, S. Mintova, *National Science Review* **2020**, *7*, 1726-1742.

[23] J. R. Cabrero-Antonino, A. Leyva-Pérez, A. Corma, *Angewandte Chemie International Edition* **2015**, *54*, 5658-5661.

[24] P. Mingueza-Verdejo, D. Velázquez-Ojeda, C. Bilanin, F. Garnes-Portolés, S. Rodríguez-Nuévalos, R. Pérez-Ruiz, J. Oliver-Meseguer, A. Leyva-Pérez, *Journal of the American Chemical Society* **2025**, *147*, 33256-33263.

[25] A. Feliczak-Guzik, *Microporous and Mesoporous Materials* **2018**, *259*, 33-45.

[26] A. Corma, *Chemical Reviews* **1997**, *97*, 2373-2420.

[27] A. Beuque, M. Barreau, E. Berrier, J.-F. Paul, N. Batalha, A. Sachse, L. Pinard, *Catalysts* **2021**, *11*, 282.

[28] Y. Han, L. Du, Y. Zhu, Y. Xu, X. Bai, Y. Ouyang, Y. Luo, X. Shu, *Catalysts* **2023**, *13*, 73.

[29] E. A. Uslamin, H. Saito, N. Kosinov, E. Pidko, Y. Sekine, E. J. M. Hensen, *Catalysis Science & Technology* **2020**, *10*, 2774-2785.
